# The microbiome: regulating anti-tumor immunity

**DOI:** 10.3389/fimmu.2026.1781872

**Published:** 2026-03-17

**Authors:** Yan-Lin Tao, Xin-Xin Wu, Jing-Ran Wang, Miao Liu, Ya-Nan Liu, Yong-Qi Lian, Zhao-Yu Liang, Shu-Fen Zhu

**Affiliations:** 1 Department of Intensive Care Unit, Affiliated Hospital of Inner Mongolia Medical University, Hohhot, China; 2 Department of Intensive Care Unit, Inner Mongolia Autonomous Region People’s Hospital, Hohhot, China; 3 Department of Respiratory Medicine, Dingzhou People’s Hospital, Dingzhou, China; 4 Physical Examination Center, Affiliated Hospital of Inner Mongolia Medical University, Hohhot, China

**Keywords:** microbiome, tumor, tumor immunity, immune regulatory cells, tumor microenvironment

## Abstract

Microorganisms distributed throughout the human body have always been a complex ecosystem that has long coexisted with other organisms. They are involved in essential key links, such as nutrient absorption, energy regulation, metabolism, toxin clearance, and immune regulation. With a deeper understanding of the microbiome, many studies have shown that the microbiome is also actively involved in the occurrence and development of tumors. The core mechanism of dysregulated microorganisms and their derivatives in the treatment response and toxicity management of tumors is the regulation of the immune function. This article explores the evolution of the microbiome and its impact on the immune function during tumor progression, and focuses on analyzing cutting-edge treatment strategies targeting the microbiome, as well as future research directions and challenges in clinical translation.

## 1 Introduction

Under the global trends of population growth, aging, and changes in risk factors, tumors have become a serious threat to human survival outcomes. The incidence and mortality rates are increasing. Tumors are not merely simple clusters of cancer cells, but rather highly organized “abnormal organs” that are characterized by differences such as cancer spectrum transformation and genetic susceptibility, resulting in a huge global burden. Currently, research on big data and artificial intelligence is continuously being applied to tumor trend analysis, risk prediction, screening optimization, and precise prevention. In this context, attention is gradually increasing. The rapid popularization of microbial molecular technologies that do not rely on cultivation has laid a solid foundation for research on microorganisms. For example, technologies such as liquid biopsy and spatial omics have opened up new horizons for the application of microbial groups in the diagnosis and treatment of tumors and are also emerging hotspots. At the same time, research on the immune system in tumors is ongoing. Its complex signal transduction and composition of microenvironment components are cornerstones for the development of modern immunotherapies. Therefore, clarifying the mutual influence between the microbiome, immune system, and tumors may have practical application value in tumor prevention and prognostication, and may become potential therapeutic intervention targets for multiple tumor sources.

The relationship between microorganisms within tumors and their development has been established: (1) by inducing increased DNA damage and genomic instability to promote tumor formation, (2) by regulating chronic inflammatory responses, complement systems, etc., to affect the host immune system and either reduce or enhance tumor progression, and (3) by influencing host secreted factors and drug metabolism (1, 2). Studies have shown that the intestinal microbiome is indispensable for shaping the host immune response, and that the intratumoral microbiome may also play a key role in the local immune response within the tumor microenvironment (TME), thereby influencing tumor progression, as shown in **Table 1**.

**Table 1:** The key details and mechanisms by which microorganisms affect tumor progression.

Finding	Mechanism	Relevant factors	References
Viruses can cause cancer either by expressing oncogenes or through immune regulation.	Human tumor viruses can cause lifelong infection of the natural ecological niche and effectively evade the host’s immune system during the long-term chronic infection process.	The tumor progression is promoted by factors such as the type of tumor virus, cell susceptibility, tumor suppressive signals resembling innate immune signals, and regulation of tissue stem cell plasticity.	Regulating cellular plasticity to persist: a way for tumor viruses to triumph.
Colibactin-producing *Escherichia coli* locally supports the energy balance within tumors, and lipid overload reduces the immunogenicity of tumors.	Intestinal microecological imbalance is associated with the enhancement of new fat formation. *Escherichia coli* locally establishes a high glycerophospholipid microenvironment, reducing immunogenicity.	*Escherichia coli* causes lipid droplets to accumulate in cancer cells infected by it, leading to oxidative cleavage of DNA double-strand breaks and the formation of lipid peroxides. Colibactin-producing *Escherichia coli* may be the cause of metabolic imbalance in the progression of colorectal cancer.	The colibactin-producing Escherichia coli alters the tumor microenvironment to immunosuppressive lipid overload facilitating colorectal cancer progression and chemoresistance.
*Escherichia coli* synthesizes PKs, which produce colibactin, promoting rectal carcinogenesis.	Bacillus subtilis alkylated DNA induces double-strand breaks in the genome.	It is related to tumor classification, lymph node metastasis, and the abundance of *Escherichia coli.*	Pks-positive *Escherichia coli* in tumor tissue and surrounding normal mucosal tissue of colorectal cancer patients.
*Fusobacterium nucleatum* induces Wnt/β-catenin regulatory molecule claudin to promote colorectal cancer.	FadA and E-cadherin induce the expression of membrane-associated protein A1, and they form a complex with β-catenin in cancer cells, activating the β-catenin signaling pathway, which leads to the overexpression of cancer genes such as cyclin D1 (CCND1).	It is related to the proliferative nature of cell carcinogenesis, tumor type, expression of membrane protein A1, and *Fusobacterium nucleatum*, and jointly promotes colorectal cancer.	*Fusobacterium nucleatum* promotes colorectal cancer by inducing Wnt/β-catenin modulator Annexin A1.
*Fusobacterium* promotes the development of colorectal cancer by activating the Wnt/β-catenin signaling pathway through Cdk5.	*Fusobacterium* directly enhances the expression of CdK5, and the activated Wnt/β-catenin signaling pathway promotes the proliferation of CRC cells, exerting an effect on the proliferation of SW480 cells.	The abundance of *Fusobacterium*, cell carcinogenesis, high expression of Cdk5, and activation of Wnt/β-catenin signaling pathway.	*Fusobacterium nucleatum* Promotes the Progression of Colorectal Cancer Through Cdk5-Activated Wnt/β-Catenin Signaling.
HPV E5 stimulates excessive proliferation of keratinocytes by upregulating the EGFR signaling pathway.	The HPV-E5 protein promotes the phosphorylation and activation of EGFR on the cell surface, enhances cell proliferation and metabolic activity, and facilitates the early gene expression of the virus.	HPV has strong adaptability for survival, significant carcinogenic potential of E5, and expression of EGFR signaling pathway.	Impact of HPV E5 on viral life cycle via EGFR signaling.

## 2 Induction of DNA damage

### 2.1 Produce genotoxic substances

**Figure 1:**
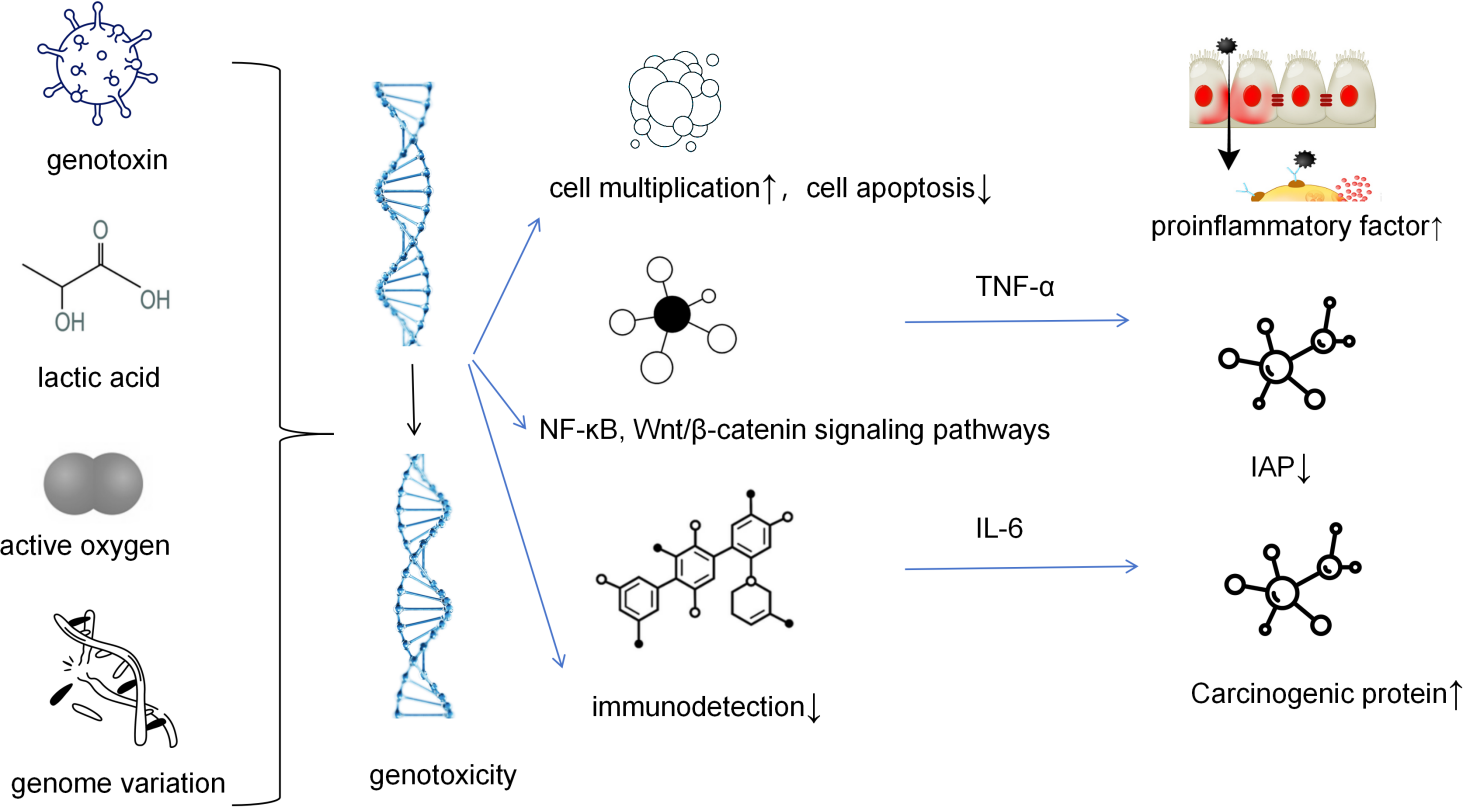
IAP, inhibitor of apoptosis protein. Microorganisms (such as certain strains of Escherichia coli) produce gene toxins such as colibactin, which can directly cause DNA double-strand breaks. This leads to increased genomic instability and is the “initial driving force” for tumor formation. Microorganisms (and cancer cells themselves) metabolize to produce large amounts of lactic acid, creating an acidic microenvironment. This acidic environment not only directly promotes cell proliferation but also inhibits the function of immune cells and provides conditions for epithelial-mesenchymal transition. Microbial infection or metabolic activities induce cells to produce a large amount of reactive oxygen species, which act as a key second messenger, activating signaling pathways such as NF-κB. Activated NF-κB enters the cell nucleus and initiates the transcription of pro-inflammatory factors and anti-apoptotic proteins, jointly creating a microenvironment that promotes cell survival, proliferation, and immune evasion.

Microorganisms can produce toxins that damage DNA. *Escherichia coli* carries a gene island named pks (polyketide synthase), which can synthesize colibactin to alkylate the host cell’s DNA and directly cross-link with the DNA molecule, thus causing double-strand DNA breaks (3, 4). During an attempt to repair the damage, the probability of errors is extremely high, resulting in cell cycle arrest and aging, and thus causing genetic mutations. In addition, *Campylobacter jejuni* produces cytolethal distending toxin (CDT), a gene toxin with DNase activity that can cause double-stranded DNA breaks (5, 6). Eventually, this leads to molecular changes and tumor transformation, thereby promoting tumor growth, invasion, and metastasis (**Figure 1**). The genus *Fusobacterium* produces fissile lysin (BFT), a protein hydrolytic toxin that can disrupt intestinal tight junctions, increase their permeability, and trigger pathways such as NF-κB and MAPK after damage, thus allowing the carcinogenic inflammatory cascade to participate in the carcinogenic process (7). It can also induce reactive oxygen species (ROS) bursts, causing oxidative DNA damage related to the B. fragilis toxin (bft) gene.

### 2.2 Participation in the inflammatory response

*Fusobacterium nucleatum* induces the expression of Annexin A1 through the FadA adhesion protein and activates the Wnt/β-catenin signaling pathway, resulting in the nuclear translocation of β-catenin, the overexpression of Wnt genes and cancer genes c-Myc, and cell cycle protein D1 (CCND1), which stimulates the growth of cancerous colorectal cells (8). In addition, *Fusobacterium nucleatum* upregulates Cdk5 to induce Wnt/β-catenin signaling transduction to promote colorectal cancer proliferation (9), while activating the β-catenin signaling pathway through FadA and Fap2, and inhibiting the anti-tumor immune response to promote cancer proliferation (3). It can also promote tumor growth in CRC in a TLR4-dependent manner, with its effect involving the activation of the IL-6/p-STAT3/c-MYC signaling pathway (10), thus forming a positive cycle that leads to chronic inflammation and also promoting tumor progression.

### 2.3 Shaping tumor immunity

The Warburg effect refers to the way cancer cells obtain ATP through the glycolytic pathway of oxidation. This energy acquisition method is inefficient but its rate is rapid (11). The generation of a large amount of lactic acid was also evident. This does not exclude the possibility that the mitochondrial activity has been saturated, which inhibits the activity of natural killer(NK) cells substances and cytotoxic T cells under acidic conditions and affects the chemotactic migration of neutrophils and dendritic cells (12), thus directly inhibiting the immune function. This adaptive advantage increases the success rate of tumor cells evading immune surveillance and simultaneously promotes ROS generation, thereby leading to increased cell proliferation and the inhibition of apoptosis (13). In the body, cell division and proliferation are accelerated to adapt to such damage. The possibility of replication mutations gradually increases due to oxidative damage of reactive oxygen species. The human papillomavirus genome undergoes variation and it is integrated into the host cell genome (14–16), which can directly target host cell E2 and other anti-cancer proteins (17). The DNA repair program fails to start, thus inducing cell apoptosis, while the ability of p53 protein to clear DNA damaged cells decreases, thus facing the continuous expression of viral oncogenes E6 and E7, eliminating cell cycle checkpoints, and inhibiting immune detection. These various mutations accumulate continuously (18), ultimately leading to cervical cancer.

These results indicate the ability of the microbial community to regulate cell proliferation and metastasis. In pancreatic ductal adenocarcinoma, MBL in the tumor environment can recognize the fungal cell wall polysaccharides of *Malassezia*, activate C3 convertase, and promote cell proliferation, migration, and invasiveness (19, 20). None of these studies suggest that microbial recognition plays a promoting role in a certain “tumor-cancer” model and it also exerts a regulatory effect on the transcription of target genes (see **Table 1**).

### 2.4 Treatment strategies for DNA damage

Genomics reveal that most human cancers contain microorganisms within the tumors, the types of which are related to the cancer type. Most are distributed in ecological niches with immune and epithelial functions, facilitating the progression of cancer and influencing the treatment response of patients (21). The prognosis of right-sided colorectal cancer is poor, and its therapeutic effect is also poor. This may be related to the involvement of tumor-associated microorganisms in chemotherapy resistance and tumor recurrence (22). Therefore, blocking the synthesis of colibactin by Pks-positive *Escherichia coli* or directly neutralizing it without affecting the balance of the microbiota may become a research frontier in the future. During anti-tumor treatment, radiotherapy and chemotherapy drugs cause DNA damage in tumor cells, thereby inhibiting the progression of tumors. Therefore, it is particularly important to effectively prevent DNA repair in tumors. In addition to microbial intervention therapy, which is currently a hot topic in the medical research field, Warburg inhibitors are also promising anti-tumor strategies. Chen et al. (23) demonstrated that antagonizing the lactate produced under the Warburg effect can interfere with DNA repair, and using the LDHA inhibitor stiripentol and genotoxic therapies, such as cisplatin and radiation agents, has a synergistic effect in improving the patient prognosis. However, whether there are any unknown effects and how to precisely target off-target toxicity and convert preclinical efficacy into reliable clinical benefits remain significant challenges. In the future, it may be necessary to integrate the tumor metabolic characteristics of patients and microbial evidence to formulate more precise multi-pathway combined blocking strategies (24).

## 3 Participation in tumor immunity

**Figure 2:**
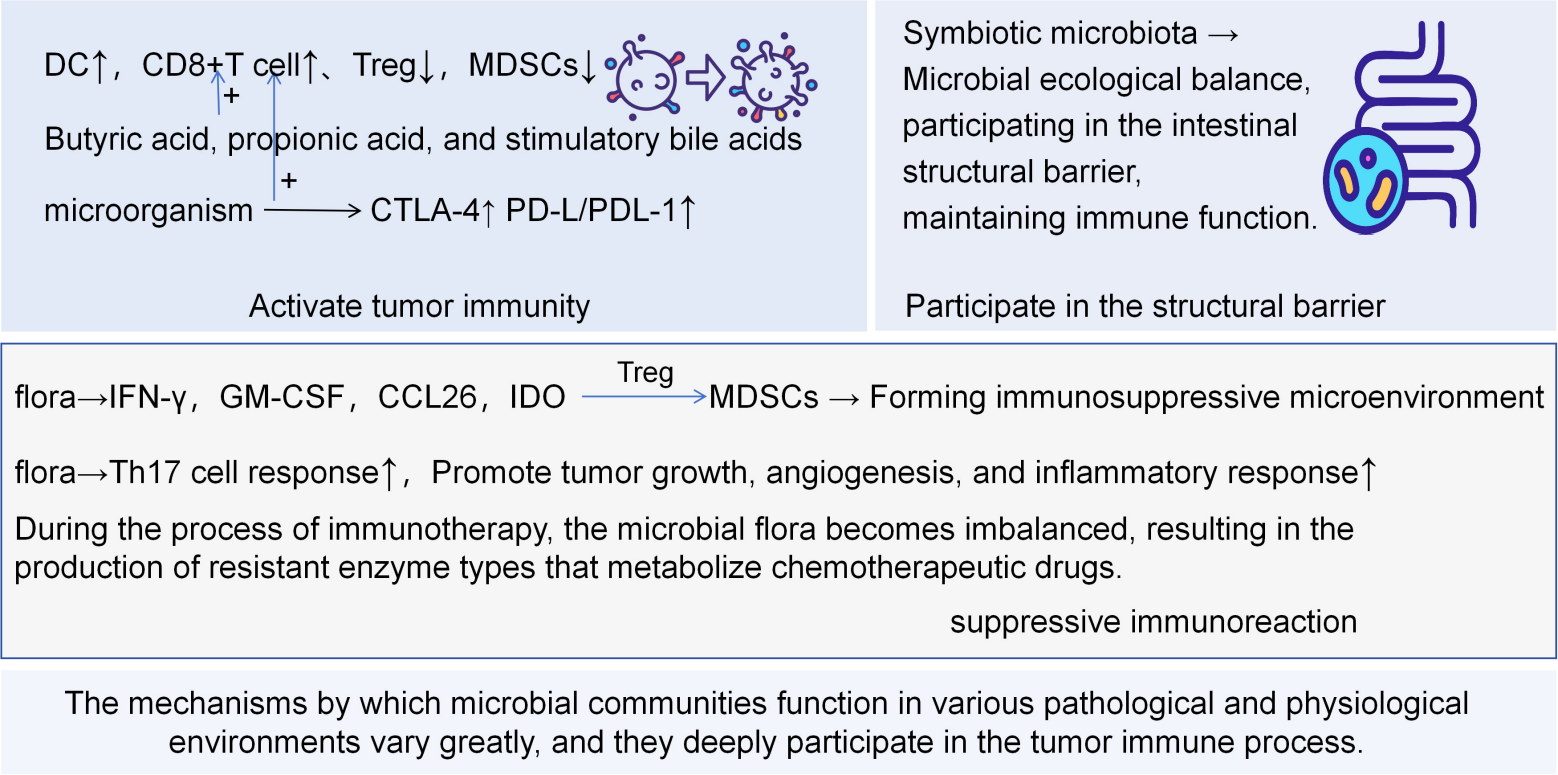
DC, Dendritic Cell; Treg, Regulatory T Cell; MDSCs, Myeloid-derived Suppressor Cell; GM-CSF, Granulocyte-Macrophage Colony-Stimulating Factor; IDO, Indoleamine 2,3-Dioxygenase and Extracellular Enzyme that Produces Adenosine. Some bacterial antigens or DNA fragments can be captured by dendritic cells, promoting their maturation, thereby more effectively cross-presenting tumor antigens to cytotoxic T cells. The bacterial components can act as ligands for pattern recognition receptors and directly activate immune cells, creating a pro-inflammatory tumor microenvironment, enhancing immune surveillance, and specifically, certain metabolites can directly enhance the killing function of T cells or promote T cell infiltration into the tumor core through regulating chemokines. On the contrary, specific pathogenic bacteria secrete chemokines to recruit suppressive cells such as myeloid-derived suppressor cells and regulatory T cells to the tumor microenvironment, directly inhibiting the activation and proliferation of T cells. Additionally, expressed outer membrane proteins can directly bind to immune inhibitory receptors on T cells, forcibly shutting down the killing function. The persistence of bacteria can lead to chronic inflammation, releasing factors that promote cell proliferation and angiogenesis, indirectly inhibiting specific immunity, and facilitating tumor progression. Maintaining the integrity of the intestinal epithelium, inhibiting pathogen colonization, and participating in the structural barrier function effectively prevent microorganisms and their products from entering the circulatory system, thereby avoiding systemic chronic inflammation. In summary, the microbiome regulates the balance between tumor immune surveillance and immune escape from three levels - initiation of killing, establishment of inhibition, and maintenance of homeostasis - through the “microbiota-metabolite-immune” axis, providing new targets for tumor prevention and treatment.

The microbial flora, particularly the gut microbiome, has been proven to act as a key regulator of the immune system and participate in the regulation of tumor occurrence, development, and treatment (25). Traditionally, the association between microorganisms and cancer has mainly focused on direct carcinogenic pathogens (such as HPV, Helicobacter pylori, hepatitis B virus, and hepatitis C virus) (13, 26). However, the advancements in metagenomics research have revealed that the vast microbial community that is symbiotic within the human body exerts systematic immune regulation, which indirectly and powerfully influences the progression and treatment outcome of cancer (27) (see **Figure 2**), playing a crucial role in the immune cycle of different types of tumors.

### 3.1 Activation of tumor immunity

Tumor immunotherapy works by removing the inhibition of tumors on the immune system and activating the body’s antitumor immunity. It has been confirmed that in the highly diverse tumor microbiome, neutrophils, NK cells, macrophages, and regulatory T-cells (Tregs), the innate immune cells, are widely enriched and are associated with the activation of epidermal growth factor receptor (EGFR), transforming growth factor β (TGF-β), and TNF-α signaling pathways, and involve significant enhancement in the expression of extracellular matrix (ECM) tissues and antimicrobial peptide pathways (28).

#### 3.1.1 Activation of immune cells

Microbiome or its components (such as LPS) can stimulate pattern recognition receptors (such as Toll-like receptor, TLR) on dendritic cells (DCs), enhancing their maturation and antigen-presenting capabilities. Through lymphatic vessels, they can migrate to the tumor-draining lymph nodes to activate T-cells. Activated DCs can more effectively cross-present tumor antigens to cytotoxic CD8+ T-cells, initiating specific killing responses. Lin et al. (29) discovered a novel intestinal bacterium of the Ruminococcaceae family named YB328, which significantly enhanced the antigen-presenting ability and co-stimulatory signals of DCs by activating the TLR7/9-MYD88 pathway. Its abundance is positively correlated with the infiltration levels of dendritic cells and CD8+ T-cells within the tumor, especially the differentiation of CD103^+^CD11b^-^ cDCs. It induces the migration of intestinal DCs to the TME, activates tumor-specific CD8+ T-cells *in situ*, and induces the expression of PD-1, thereby enhancing the antitumor immune response and sensitivity to immunotherapy. *Akkermansia* muciniphila and pathogenic bacteria-derived endotoxins can activate immature DCs to maintain immune responses, and their regulation of the ability of DCs to activate γδ T lymphocytes has little effect (30), while γδ T-cells are considered a natural host defense and effective MHC unrestricted effector factors for anti-tumor responses (31). Microbial communities such as Lachnoclostridium and Acinetobacter in the tumor can regulate chemokines CXCL9 and CXCL10 to affect CD8+ T-cell infiltration, thereby improving the survival rate of patients with cutaneous melanoma (32).

Regulatory T cells (Tregs) are the main mediators of immunosuppression. A healthy microbiota ecosystem helps to stabilize the excessive activation and proliferation of Tregs, thereby creating space for effector T cells to function. Sun et al. stated that *bifidobacteria* can optimize the composition of the mouse symbiotic microbiota under the condition of immune checkpoint CTLA-4 blockade. Through the IL-10/IL-10Rα self-stimulatory signaling loop (Self-Stimulatory Loop), it enhances the function and metabolism of intestinal regulatory T cells (Tregs), not only alleviating the intestinal inflammation induced by anti-CTLA-4 antibodies (33), but it also helps to maintain regional immune homeostasis under CTLA-4 blockade.

Tumor-associated macrophages are crucial in the immunosuppressive TME, which is related to tumor development, metastasis, and resistance to cancer treatment. Macrophages can differentiate into the M1 and M2 phenotypes. The M1 macrophage phenotype is characterized by elevated levels of pro-inflammatory mediators, such as IL-1β, IL-12, and TNF-α, as well as the induction of inducible nitric oxide synthase, reactive oxygen species, and inhibition of tumor cell proliferation. The M2 phenotype is induced by Th2 cytokines (such as IL-4 or IL-13), and is characterized by the synthesis of arginase 1 (Arg1) and the presence of inflammation zone 1 (Fizz1). These factors play crucial roles in reducing inflammatory responses, promoting wound healing, and facilitating tissue regeneration (34). Myeloid-derived suppressor cells (MDSCs) are a group of immature/abnormally polarized myeloid cells, including monocyte-type MDSCs (M-MDSCs) and neutrophil-type MDSCs (PMN-MDSCs). M-MDSCs directly promote tumor metastasis. MDSCs in the TME help tumor metastasis and immunosuppression by inhibiting T-cell proliferation and effector functions (35), remodeling the extracellular matrix (36), regulating the expression of extracellular enzymes that metabolize adenosine (37, 38), inhibiting lymphocyte homing, and inducing vascular leakage, and are key regulators of the cancer immune response (35, 39). Healthy microbiome can inhibit the recruitment and function of these key immunosuppressive cells, thereby relieving the suppression of T-cell activity.

#### 3.1.2 Direct immune regulatory effects of metabolic products

Short-chain fatty acids (SCFAs) such as butyric acid and propionic acid not only nourish the intestinal barrier but also directly enhance the stem cell-like characteristics and memory function of CD8^+^T-cells through epigenetic mechanisms (such as inhibition of histone deacetylases) and inhibit the immunosuppressive activity of Tregs. After dysbiosis of the intestinal flora, the production of SCFAs decreases and the percentage of M2-like macrophages increases. Oral supplementation of SCFAs can increase the proportion of M1-like macrophages in the TME, and during this M1 polarization process, it is possible that SCFAs achieve this by enhancing glycolysis in macrophages (40). The metabolites related to the *Clostridium* genus trimethylamine N-oxide (TMAO) have dual properties. In triple-negative breast cancer, they induce tumor cell pyroptosis by activating the endoplasmic reticulum stress kinase PERK, thereby enhancing CD8 T cell-mediated anti-tumor immunity to inhibit tumor growth (41). In colorectal cancer, they activate the Wnt signaling pathway, upregulate the expression of β-catenin protein, and promote the generation of ROS, leading to oxidative stress, making tumor cells insensitive to proliferation inhibitory signals and apoptosis, while possibly inducing DNA damage and epigenetic changes which can thus promote colorectal cancer cell proliferation and accelerate tumor growth (42).

Secondary bile acids (such as cholic acid and 3-oxidized cholic acid) produced by the metabolism of the gut microbiota, in addition to facilitating nutrient absorption, maintaining glucose homeostasis, and regulating energy expenditure, have been shown to interfere with tumor occurrence and development, which is related to the type of tumor and has a dual effect, as shown in **Table 2**. In a study on mouse colorectal cancer, it promoted the differentiation of macrophages, dendritic cells, helper T cells (Th) 17 cells, B cells, T cells, etc. (43), inhibited the Ca2+-activated T nuclear factor (NFAT) 2 signaling pathway, reduced the infiltration of CD8 + T cells (44), and promoted tumor progression. In contrast, Varanasi et al. proposed (45) that inhibiting the synthesis of secondary bile acids in hepatocytes can enhance the tumor-specific T cell response and thus reduce tumor growth and also increase the sensitivity of tumors to immune therapy targeting programmed cell death protein 1 (anti PD-1) (see **Table 2**).

**Table 2:** The environment-dependent nature of short-chain fatty acids, bile acids, and TMAO.

Description	SCFA	Bile acid	TMAO
Basic properties	Dietary fiber is fermented by intestinal bacteria to produce substances such as acetic acid, propionic acid, and butyric acid.	The primary bile acids are metabolized in the intestine to form secondary bile acids (deoxycholic acid and cholic acid)	Dietary choline/methionine is metabolized by gut bacteria into TMA, which is then oxidized by liver FMO3 to form TMAO.
Overall tumor effect	Tumor suppression as the main function: It inhibits histone deacetylases, blocks the cell cycle, induces apoptosis of cancer cells, inhibits angiogenesis and invasion/migration. At lower concentrations or in specific microenvironments, it can promote inflammation and support the proliferation of intestinal epithelial cells, with potential tumor-promoting tendencies.	It has duality: 1. Hydrophobic bile acids mainly promote tumor growth, inducing ROS oxidative stress, activating the NF-kB/Wnt pathway, and promoting inflammation, proliferation and metastasis. 2. Hydrophilic bile acids: mainly inhibit tumor growth, regulating metabolic and inflammatory balance, highly dependent on types and subtypes.	Mainly resents tumor-promoting effects: Associated with chronic inflammation, activation of cancer-related fibroblasts, promotion of thrombosis and tumor angiogenesis, and mediating metabolic disorders - malignant progression of tumors and chemotherapy resistance, etc.
Tumor type specificity	1. Colorectal cancer: Butyric acid is one of the main energy sources for intestinal cells and has protective effects. At high concentrations, it can induce apoptosis of cancer cells. 2. Immune-related tumors: Through the functions of Treg and CD8+ T cells, it may enhance the efficacy of immunotherapy. 3. Leukemia and lymphoma exhibit apoptotic induction activity, while lung cancer/gastric cancer show concentration-dependent bidirectional effects.	1. Colorectal cancer, hepatocellular carcinoma, cholangiocarcinoma: Secondary bile acids act as risk factors and can promote cancerous effects. 2. Esophageal cancer, gastric cancer: Positively correlated with exposure to reflux bile acids. Breast cancer/prostate cancer: Through the enterohepatic axis and cross-regulation with hormone metabolism, they exhibit a tumor-promoting tendency.	1. Colorectal cancer: High levels of TMAO are associated with an increased risk of CRC and poor prognosis. 2. Breast cancer/prostate cancer: High levels of TMAO are related to poor prognosis and a higher risk of metastasis. Pan-cancer: As a driver of chronic inflammation and immunosuppression.
Interaction with radiotherapy	Potential radiosensitizers: 1. They may exert a role in alleviating radiation-induced colitis by exerting anti-inflammatory, antioxidant effects, promoting epithelial cell repair, etc., and also inhibit normal tissue fibrosis induced by radiotherapy. 2. Butyric acid may make cancer cells more sensitive to radiotherapy through epigenetic regulation.	1. Hydrophobic bile acids: They affect the toxicity of radiotherapy and may aggravate the DNA damage and intestinal inflammation caused by radiotherapy, increasing the risk of radiation-induced intestinal injury. 2. Hydrophilic bile acids: They have mild radiotherapy sensitization effects and can alleviate the radio-induced inflammatory damage in the liver and intestines.	Mainly related to radiotherapy resistance: The chronic inflammatory and fibrotic microenvironment induced by TMAO may lead to radiotherapy resistance and enhance the DNA repair ability of tumor cells. Reducing the level of TMAO or inhibiting its signaling may become a new idea for enhancing radiotherapy sensitivity.
Interaction with immunotherapy	Immune regulators: SCFAs enhance the efficacy of immune checkpoint inhibitors. They can promote the function and memory formation of CD8+ T cells, inhibit the M2 polarization of tumor-associated macrophages (TAM), regulate the balance of Tregs, and improve the tumor immune microenvironment.	Dual and complex regulatory effects: 1. Immunosuppression: Hydrophobic bile acids induce T cell exhaustion, recruit M2-type TAMs, inhibit DC cell maturation, and weaken the efficacy of immunotherapy. 2. Moderate immune regulation: Hydrophilic bile acids improve the immunosuppressive microenvironment and work in synergy with immune checkpoint inhibitors. The overall effect depends on the bile acid spectrum.	Mainly has an inhibitory effect: High levels of TMAO are associated with resistance to anti-PD-L1 therapy and a decrease in the abundance of tumor-infiltrating lymphocytes. It may involve pro-cancer pathways such as PI3K-Akt-mTOR, inducing M2-type macrophage polarization, and creating an immunosuppressive microenvironment.

#### 3.1.3 Enhancement of therapeutic efficacy

At present, research on the mechanism of drug resistance generated by microorganisms within tumors during the anti-tumor process is constantly being refined. Paying attention to microorganisms within tumors during the treatment process is likely to become a key target for overcoming drug resistance in the future. For example, in the treatment of metastatic melanoma, Baruch et al. proposed that fecal microbiome transplantation enhances the activity of DCs and the CD8+ T-cell response to promote the response to CTLA-4 and PD-1/PD-L1 blocking immunotherapy and enhance the T-cell response (46, 47). In addition, Mirji et al. (48) stated that pancreatic ductal adenocarcinoma is ineffective in immune checkpoint blockade therapy, and a metabolite derived from the gut microbiome, trimethylamine N-oxide, through the type I interferon (IFN) pathway, can enhance antitumor immunity against pancreatic ductal adenocarcinoma, thereby reducing tumor growth.

### 3.2 Suppression of immune response

Dysbiosis may create an immunosuppressive environment that facilitates tumor progression. Some harmful bacterial communities may induce immunosuppressive cells (such as Tregs and MDSCs) to create a microenvironment that allows tumors to evade immune attacks. Tumor immunotherapy benefits only a small proportion of patients with tumors. In addition to individual heterogeneity and toxicity of the treatment regimen, it is crucial to clarify the underlying pathogenic mechanism.

#### 3.2.1 Induction of immunosuppressive microenvironment

The TME, mentioned multiple times in our previous description, is a complex ecosystem that tumor cells rely on for survival and development. It is composed of tumor cells, various stromal cells (such as fibroblasts, immune cells, and endothelial cells), ECM, signaling molecules (such as cytokines and chemokines), special physical and chemical conditions (such as hypoxia and acidosis), and special microorganisms. Studies have shown that the TME plays a decisive role in the occurrence and development of tumors, immune evasion, metastasis, and treatment resistance. It not only provides nutrition and support for tumors but also participates in the malignant process of tumors through complex intercellular connections. Commensal bacteria directly affect tumor occurrence, progression, and response to treatment (1). The immune system plays a dual role in the occurrence and development of tumors. On the one hand, it performs immune surveillance functions to identify and eliminate transformed malignant cells, exerting an anti-tumor effect. On the other hand, under the selective pressure of the tumor microenvironment, it can undergo immune editing, and by establishing an immunosuppressive microenvironment, promoting angiogenesis, and inducing epithelial-mesenchymal transition, it participates in immune escape, local invasion, and the distant metastasis of tumors. Various factors such as IL-6, IFN-γ, IL-1β, granulocyte-macrophage colony-stimulating factor, TNF-α, and vascular endothelial growth factor in the TME environment, while tumor cells express chemokine CCL26, indoleamine 2,3-dioxygenase, and extracellular enzymes that produce adenosine, and with the assistance of Tregs, MDSCs are recruited, thereby exerting immunosuppressive functions and promoting tumor growth (35). Liu et al. found that the symbiotic bacteria A. sydowii in the lungs was significantly enriched in lung adenocarcinoma, stimulating key molecules IL-1β through the β-glucan-mediated Dectin-1/CARD9 signaling pathway, thereby inducing the differentiation of MDSCs and significantly impairing the killing ability of lung adenocarcinoma cells induced by T-cells (49). Zhang et al. (50) demonstrated that intestinal gram-negative bacteria/LPS, through TLR4-dependent chemokine CXCL1 production, induced the accumulation of CXCR2+ PMN-MDSCs and controlled the formation of an immunosuppressive microenvironment in liver cells, thereby promoting liver tumor growth, once again confirming that the intestinal microbiome is an important regulator of anti-tumor immunity. Chronic or specific microbial stimulation may lead to the continuous high expression of inhibitory receptors such as PD-1 and CTLA-4 on immune cells, causing T-cells to be in a depleted state and unable to effectively kill tumor cells (51).

#### 3.2.2 Promotion of chronic inflammation

This is the most important and widespread mechanism by which microorganisms promote cancer development. Certain pathogenic bacteria or dysbiosis may trigger excessive Th17 cell response. The pro-inflammatory factors secreted by Th17 cells, such as IL-17, can create an inflammatory environment conducive to tumor growth, angiogenesis, and metastasis (2). Jin et al. proposed that symbiotic bacteria in the lungs stimulate myeloid cells to produce Myd88-dependent IL-1β and IL-23, inducing the proliferation and activation of Vγ6+Vδ1+γδT-cells, thereby generating IL-17 and other effector molecules, promoting inflammation and the proliferation of tumor cells (27).

We previously described that pathogenic bacteria produce genotoxins that cause an increase in DNA double-strand breaks in the host, thereby increasing the frequency of gene mutations and driving tumor formation. Researchers have shifted their focus to multiple aspects of microbial community metabolism. Guo et al. stated that Fusobacterium nucleatum is a common bacterial strain in colon cancer, which can stimulate tumor cells to produce extracellular vesicles rich in miR-1246/92b-3p/27a-3p and CXCL16/RhoA/IL-8. These vesicles, which are rich in unique RNA and proteins, can increase the migratory ability of infected cells to uninfected cells, thereby achieving tumor metastasis (52). Activation of the Wnt/β-catenin pathway within the tumor and loss or mutation of PTEN have been proven to lead to a deficiency in T-cell infiltration into the TME and resistance to checkpoint blockade immunotherapy (53). Butyrate can alleviate diarrhea caused by intestinal inflammation, but can also reduce the efficacy of ionizing radiation in treating cancer. Yang et al. (47) reported that local butyrate blocks the phosphorylation of TBK1 and IRF3, while inhibiting the activation of dendritic cells by STING, thereby eliminating the tumor-specific cytotoxic T-cell immune response induced by ionizing radiation. This may be a reminder that intestinal microbiota therapy should be a precise treatment, and that it needs to achieve a dynamic balance of efficacy in various diseases.

#### 3.2.3 Weakening the efficacy of cancer treatment

Researchers and medical professionals have continuously proposed and updated treatment plans in various oncology research fields. However, the lengthy research process is always accompanied by drug chemotherapy resistance, which undoubtedly increases the difficulty of treatment. The survival rate of patients with pancreatic ductal adenocarcinoma is extremely low, and despite active treatment, a high recurrence rate remains. Interventions based on bacteria in the TME have become particularly urgent. The chemotherapy regimen of placing a biliary stent combined with gemcitabine and paclitaxel promotes the growth of Enterobacteriaceae in the tumor area. Nalluri et al. speculated that this may be the reason for chemotherapy resistance (54). Additionally, Geller et al. (55) proposed that the development of resistance during the treatment of pancreatic ductal adenocarcinoma with gemcitabine was mainly related to the Gammaproteobacteria bacterial group. These intratumoral bacteria metabolize gemcitabine, rendering it inactive. Cytidine deaminase, expressed by γ-Proteobacteria, can also completely metabolize gemcitabine (2), thereby mediating resistance.

Resistance to immune therapy is a notable example. Multiple studies have confirmed that a dysregulated microbiome cannot effectively activate the required immune response, leading to treatment failure (56, 57). Chemotherapeutic agents, such as 5-fluorouracil and cyclophosphamide, increase the proportion of pathogenic Firmicutes (such as Staphylococcus) and Proteobacteria (such as Escherichia coli and Pseudomonas), while the symbiotic Bacteroidetes decreases.

These bacterial phyla that promote inflammatory responses trigger immune responses, such as tumor progression, inflammatory responses, and the ecological imbalance caused by cancer treatment, and enter a recurring vicious cycle (13). Riquelme et al. (58) proposed that antibiotic bacterial ablation in a mouse model of orthotopic pancreatic ductal adenocarcinoma reshaped the local TME, induced T-cell activation, improved immune surveillance, and increased sensitivity to immunotherapy. This may suggest a potential impact of the local microbiome on the immunotherapy process.

### 3.3 The role of the microbiome in the structural barrier function

The symbiotic microbiome is closely attached to the surface of epithelial cells to occupy potential binding sites, compete for nutrients with pathogenic bacteria, secrete antibacterial and other chemical substances and metabolic substances, and induce protective immune responses to maintain the integrity of the barrier and prevent the translocation of bacteria and their metabolites into the bloodstream (59). Through this multidimensional, collaborative approach, a dynamic, intelligent, and efficient defense system is constructed, which is the foundation for ensuring the integrity of the barrier function and overall health. Barrier disruption can lead to a series of systemic inflammatory responses, which is a driving factor for many cancers, as shown in **Table 3**.

**Table 3:** Microbial flora and mucosal barrier.

Different aspects	Role of the microbiome	Effect mechanism	Impact on various barriers
Structural level	Strengthen connections, promote repair	Proteoglycans, short-chain fatty acids	Enhance tight junctions between epithelial cells
Physical level	Occupation, competition	Formation of biofilms, nutrient competition	Prevent pathogen colonization
Biochemical level	Antibacterial, shaping microenvironment	Bacteriocins, lactic acid, SCFAs	Inhibit tumor growth
Immune level	Immune regulation	Treg cells, SlgA, cytokines	Form immune tolerance, control inflammatory response

Wu et al. showed that Fusobacterium nucleatum attaches to intestinal epithelial cells through the FadA protein, binds to vascular endothelial cadherin to alter endothelial integrity, and then stimulates the activation of oncogenic miR21, leading to overexpression of TLR-4/nuclear factor-κB and promoting tumor development. Once colorectal tumors progress, the Fap2 lectin binds and adheres to tumor cells expressing Gal-GalNAc and then enriches Fusobacterium nucleatum to form a vicious cycle. Moreover, the imbalance in the intestinal microbiome caused by the enrichment of Fusobacterium nucleatum drives the dominance of the Fusobacterium genus, which undoubtedly exacerbates tumor progression (60). At the same time, Zhao et al. also emphasized the crucial role of microbial community balance in the integrity of intestinal tissues in the colonization of pathogenic bacteria. In this context, Parvimonas micra was isolated at a relatively high abundance from the feces of colorectal cancer patients. After feeding mice with this strain, they observed a high colorectal tumor burden, which was related to the differentiation of CD4+ T-cells into Th17 cells and enhancement of the oncogenic Wnt signaling pathway (61). Therefore, it can be seen that the microbiome can play a role in various stages of tumor development by regulating the host’s immunity and TME. Deeply regulating the microbiome or using it as a biomarker for improving cancer treatment may become a mainstream direction in the future.

## 4 Microbial therapeutic targets

**Figure 3:**
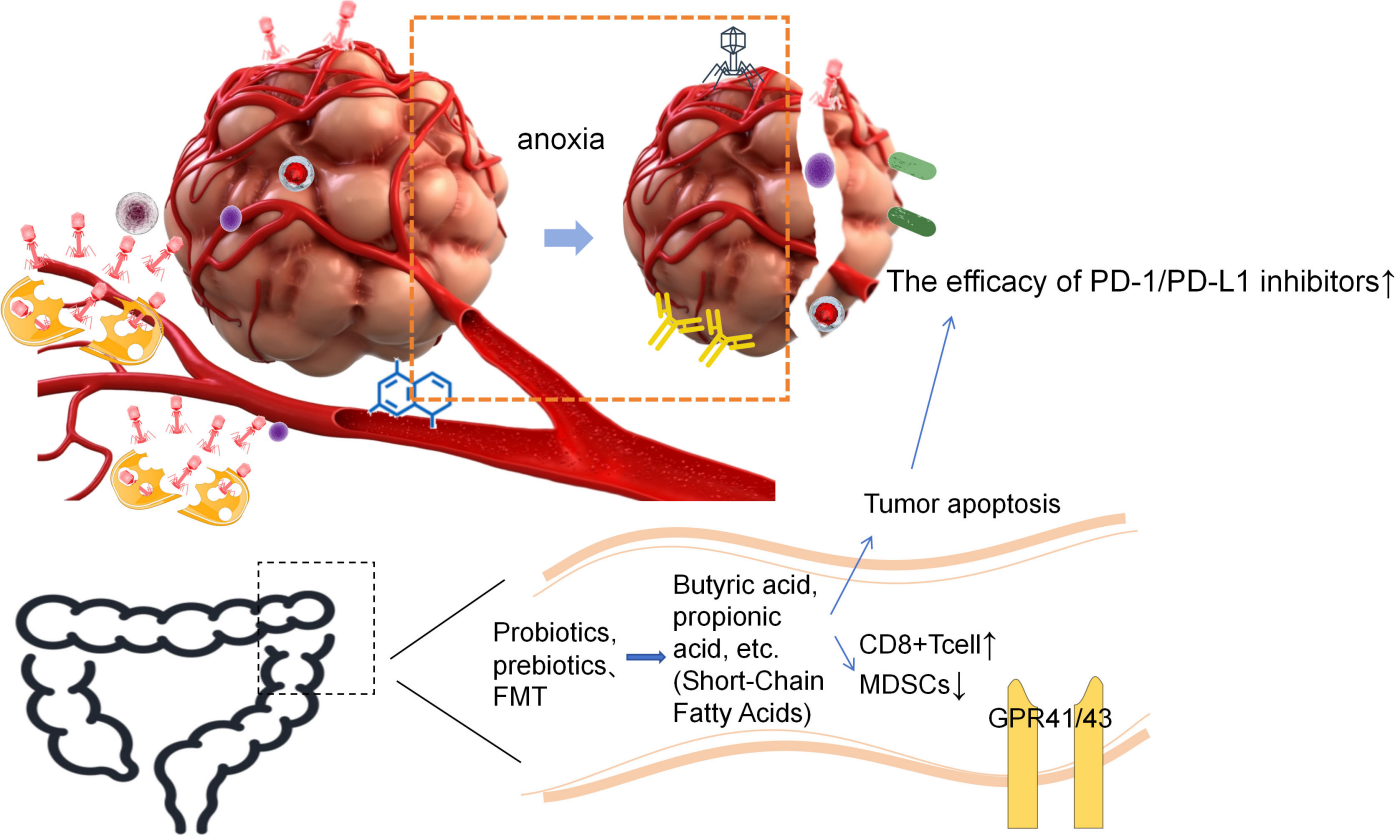
Oncolytic viruses, engineered bacteria, etc. target and gather around the tumor, lyse tumor cells, release immune factors and cytotoxic substances in situ, express antibodies, and destroy tumor cells. The microbiome in the local microenvironment can also consume essential metabolic substances, disrupt the tumor-supporting microenvironment; by enhancing the efficacy of immune checkpoint inhibitors and other pathways, it can enhance the immune treatment response, supplement probiotics, prebiotics or perform FMT to regulate the intestinal microbiome, activate the immune system. This figure summarizes the direct killing of tumor cells by microorganisms, the destruction of the tumor-supporting environment, the activation and recruitment of immune cells, the remodeling of the immunosuppressive microenvironment, revealing that microorganisms have multi-dimensional and multi-level anti-tumor effects, demonstrating the important potential of microbial therapy in comprehensive tumor treatment, providing new ideas for combined treatment and overcoming drug resistance.

Research on the association between microbiome and cancer has elevated the microbiome from an observer to an indispensable key player in cancer treatment. Current studies have confirmed that the composition and structure of the microbiome can enhance the response rate of tumor immunotherapy, alleviate the toxic adverse manifestations during treatment, provide a new perspective on predictive biomarkers, enhance the efficacy of existing therapies, especially immunotherapy, and identify future innovative treatment targets. Intervention in the microbiome to improve cancer treatment has become a highly promising new frontier, as shown in **Figure 3**.

### 4.1 Prediction and enhancement of immune therapy efficacy

Sequencing methods, such as DNA or RNA sequencing, have clearly demonstrated that tumors often accompany the enrichment of certain microorganisms (1). For instance, *Helicobacter pylori* is commonly associated with gastric cancer, and HPV is linked to cervical cancer. These results demonstrate the promoting role of the microbiome in the carcinogenesis of tumors and their development. After the use of antibiotics to reestablish the microecology of mice, a lower immune treatment response was observed (62). When using the MNZ antibiotic lineage, which is sensitive to multiple drug-resistant *fragile bacilli*, the intestinal microbiome is disrupted, thereby affecting the ability of bacteria to kill the symbiotic microbiome required for chemotherapy in tumors (63).This led to the proposal that the composition of intestinal microorganisms affects the response to chemotherapy through immunity, resulting in the division of patients into responders and nonresponders. Subsequently, researchers gradually narrowed down the range of thousands of microorganisms and have now been able to use them as predictive biomarkers for immune treatment responses (26). The microbiome affects the immune response to immunotherapy by shaping the immune status both systemically and locally in the tumor. Recent research has found that patients who do not respond to immune checkpoint inhibitors, such as those targeting programmed cell death protein (PD-1) and its ligand (PD-L1), as well as cytotoxic T lymphocyte antigen 4 (CTLA-4), usually have specific microbial characteristics (64). Miller et al. (58) demonstrated that microorganisms such as Akkermansia muciniphila and Faecalibacterium prausnitzii can generate a stronger immune response during anti-PD-L1 drug treatment, maximizing immunotherapy efficacy. An increase in genera such as Bacteroides fragilis, Burkholderia cepacia, and Faecalibacterium can increase the treatment effect in patients treated with CTLA-4-based therapy (65). The above two immunotherapy regimens enhance the immune efficacy by strengthening the tumor’s toxicity effect on the body’s own immune system T-cells through the microbiome, thereby prolonging the survival of patients and proving that the enrichment of related groups is associated with better responses to immunotherapy and survival benefits (66). Of course, the selection of treatment microorganisms, the timing of use, the relative abundance of local microecology, and patient efficacy still need to be jointly determined through multimodal combinations such as metabolomics, genetic genomics, and immunology. We need to be aware of the role of microbial communities in tumor occurrence and progression in different body parts.

### 4.2 Targeting the microbiome for cancer treatment

Exploring the microbial communities in other parts of the body besides the intestines: The microbiome within tumors, the oral cavity, and lungs are also closely related to the occurrence and treatment of local cancers and are emerging hotspots. From basic research to clinical translation, evidence is gradually improving, providing new strategies to overcome resistance to immune therapy. The microbiome has specificity and individual heterogeneity, and its mechanism of action may be precise down to the strain level rather than just the species level. The microbiome has specificity and individual heterogeneity. Its mechanism of action may be precise down to the strain level, rather than just the species level. This increases the complexity of the research. Everyone’s microbiome is unique and is influenced by multiple factors such as genes, diet, and environment (67). It is difficult to formulate a “one-size-fits-all” solution, and it is also prone to being interfered by technical traps. Currently, microbial-based tumor immunotherapy mainly includes fecal microbiome transplantation, probiotics, and prebiotics, as well as phage and live biological drug therapies.

#### 4.2.1 Fecal microbiota transplantation

Transplanting the complete gut microbiome community system of the responder into the patient’s intestine to achieve rapid microbial group remodeling, thereby changing the host’s immune state, is currently a direct and well-evidenced microbial group intervention method. Fecal microbiota transplantation (FMT) has achieved great success in treating recurrent Clostridioides difficile infections and has continued to advance in later clinical studies (68). Preliminary clinical trials have shown that transplanting the fecal microbiota of melanoma patients who respond to PD-1 treatment in patients with primary resistance can reverse their resistance status, allowing some patients to undergo microbial group remodeling and achieve clinical responses. These studies provide proof-of-concept for the clinical application of FMT (69–71). However, the main challenges lie in the standardization of donor selection, preparation of the formulation, and long-term safety. We believe that this method is more likely to be used in specific resistant populations. The combination of FMT and immune checkpoint inhibitors is feasible in the short term, but long-term safety still needs to be considered, including changes in immune toxicity and potential infection risks, such as the spread of pathogens and occurrence of unknown long-term side effects.

#### 4.2.2 Probiotics and prebiotics

We mentioned in previously that the genera Akkermansia muciniphila and Faecalibacterium prausnitzii promote the activation of immune cells in the TME, enhancing the efficacy of immune checkpoint inhibitors, or activating immune cells by releasing metabolites (such as indole-3-aldehyde), thereby improving the anti-tumor immune response. Moreover, probiotics may help maintain or improve the structure of the intestinal flora, reduce harmful bacteria, promote the growth of beneficial bacteria, and indirectly affect the efficacy of immunotherapy (59). They can alleviate adverse reactions such as intestinal dysfunction, diarrhea, and oral mucositis caused by tumor treatment and improve the quality of life of patients. Although commercial probiotics are widely used, their role in tumor immunotherapy remains controversial. Some studies have found that there is a possibility of uncertain efficacy after treatment (57, 72). Therefore, more precise clinical trials targeting specific strains are needed rather than simply using broad-spectrum probiotics. In addition, we focused on precisely improving their safety and effectiveness. Zhang et al. (73) proposed engineered bacteria based on the interaction between bacteria and the host, which can optimize targeting accuracy based on the characteristics of the TME and molecular interactions, and can construct genetically programmed and controllable expression, providing a core design strategy for engineered bacterial immunotherapy, promoting safer, more precise, and intelligent cancer immunotherapy in clinical applications. Prebiotics are indigestible food components that selectively stimulate the growth or activity of beneficial bacteria in the intestine, generating metabolites that stimulate the maturation of dendritic cells and the activation of cytotoxic T lymphocytes to stimulate the immune system (74). Pectin has a similar activity to prebiotics and can increase the abundance of beneficial bacteria such as lactobacilli in the intestine, enhance the efficacy of PD-1 in inhibiting cancer, and its derivatives can also inhibit prostate cancer, colon cancer, and cell apoptosis by inducing cell cycle arrest (75, 76). Encourage patients to adopt a diversified dietary pattern rich in dietary fiber, which is low in cost, safe, and may benefit them. However, current prospective dietary intervention studies are needed to determine prebiotic protocols.

#### 4.2.3 Live biotherapeutic products

This refers to single or composite bacterial strains that have been rigorously screened and scientifically verified and are designed to form a bacterial strain group with clear immunomodulatory functions for drug development (77, 78). For example, the Clostridium butyricum MIYAIRI 588 strain (CBM588) regulates the anti-tumor CD8+ T cell response, induces the release of tumor necrosis factor-related apoptosis-inducing ligand by polymorphonuclear neutrophils, and increases the relative abundance of beneficial bacterial communities, such as bifidobacteria, thereby enhancing the chemotherapy effect and exerting a significant anti-tumor effect (79). Additionally, Lactococcus lactis GEN3013 regulates tumor angiogenesis and directly induces cancer cell death, enhancing the therapeutic effect of PD-1 inhibitors to inhibit tumor growth (80). SER-401 (containing strains such as Faecalibacterium prausnitzii) has entered clinical trials to enhance the response of melanoma patients to immunotherapy (81). Safety needs to be strictly controlled (82), and technical barriers need to be overcome to break through genetic modification to regulate bacterial growth, ensure treatment safety, and establish a standardized detection and data analysis processes. However, this also represents the future direction of microbiome therapy standardization, mass production, clear mechanisms, and controllable quality. The challenges lie in the long drug development cycle, high costs, and need to confirm its superiority or differentiated advantages over FMT (83, 84).

## 5 Technical traps and validation in the study of the tumor microbiome

In summary, research on the intratumoral microbiome in oncology is constantly increasing, and it is also enhancing our understanding of the initiation, progression, treatment, and prognosis of cancer, thus adding new targets for cancer treatment, and achieving a shift from single-site to multi-target approaches. This is the direction we will focus on in the future. Simultaneously, technical challenges such as extraction, separation, and purification during the period from sampling to obtaining clear results, as well as issues such as the misjudgment of background bacteria and research methods, should not be underestimated. Therefore, identifying key technical pitfalls and establishing strict validation processes can provide convincing biological discovery results and ensure the accuracy of clinical translation (**Table 4**).

**Table 4:** Probiotics, prebiotics, FMT, and living biological therapy comparison.

Description	FMT	Probiotics	Living biological therapy
Positioning	Overall ecosystem transplantation Advantages: Strong effect, sufficient evidence Disadvantages: Reliance on donor species, potential safety risks.	Add a single/limited strain of bacteria, Advantages: Easy to use, low cost Disadvantages: Inconsistent therapeutic effects, the specific strain needs to be clarified.	Precisely defined microbial drugs. Advantages: Clear mechanism, controllable quality. Disadvantages: Long research and development cycle, high cost.
Patient selection	The primary indication is recurrent/refractory Clostridioides difficile infection, and patients with ICI-refractory/resistant conditions. Immune-related colitis is an exploratory indication.	The indications are quite broad: it can be used to regulate the intestinal microecology in patients with diarrhea and tumors, and also to prevent ICI-related diarrhea/colitis.	The lack of certain specific functional bacteria has led to the majority of approved/investigating products targeting ICIs in combination with anti-tumor and IBD treatments, and verifying the immune-enhancing mechanisms of specific microorganisms or their metabolites.
The relationship with ICI time	1. Early intervention with FMT should be carried out within 1–2 weeks before ICI administration or after the occurrence of immune-related adverse reactions (irAEs); 2. Avoid transplantation within 24 hours after ICI infusion to reduce interference from immune fluctuations; 3. Start ICI administration 1–2 weeks after FMT.	1. Start simultaneously with ICI treatment and take it daily for a long period; 2. Some protocols suggest delaying the administration of ICI by several hours and preloading it 1–2 weeks before the administration of ICIs.	1. Pre-treatment before ICI therapy, or used concurrently with ICI, or sequentially in combination, but avoid administration on the same day. The interval should be more than 48 hours. 2. Carrying specific drug-engineered bacteria: The timing is designed based on pharmacokinetics.
Safety considerations	1. Infection: Despite strict screening, there is still a risk of transmission and repositioning of unknown pathogens. 2. Immunity: It may induce or exacerbate immune-related adverse events (irAEs), and the donor microbiome may carry autoimmunity susceptibility traits. 3. Long-term ecology: The long-term effects of the donor microbiome on the host’s metabolism and other multiple systems are unknown. 4. In-depth screening of donor’s drug resistance genes, virulence factors, etc. is required. Assessment of the patient’s intestinal condition is also necessary. Long-term follow-up is conducted to monitor for delayed immune events, metabolic changes, new infections, etc.	1. Microbiome: Causes unexpected effects on the host’s original microbiome. 2. Immunity: Risk of excessive stimulation or interference with normal immune response. 3. Metabolism: May affect the bile acid metabolic pathway. The structure of the host’s native microbiome needs to be monitored, and it is necessary to determine whether the strains carry drug resistance genes. 4. It is necessary to monitor the structure of the host’s native bacterial community and determine whether the strains carry drug resistance genes.	1. Risks related to engineered bacteria: The potential for horizontal gene transfer of engineered bacteria, their survival and evolution in the environment, as well as exogenous infections and infusion reactions. 2. Off-target effects: The immune activation effect may trigger unexpected systemic inflammation or autoimmunity. 3. Colonization resistance: Whether it can successfully colonize in the host body. 4. Strictly conduct tests for viable bacterial counts, verification of colonization safety of strains, immune activation, and long-term genetic stability detection, and carry out strict safety endpoint evaluation.
The relationship with clinical relevant endpoints	In the efficiency/therapeutic studies, attention is paid to the progression-free survival period, the success rate and persistence of donor bacterial colonization, as well as the remission rate and recurrence rate of ICIs colitis.	Improvement in gastrointestinal symptoms, changes in specific metabolites, systemic inflammatory markers, intestinal barrier function markers, as well as the control rate of ICIs treatment and the incidence of irAEs.	Rapid enhancement of efficacy or objective response rate during the biological distribution/colonization period, overall survival, LBP colonization at the tumor site and its targeting nature, as well as changes in the concentration of target metabolites or immune mediators in the blood.

With the development of next-generation sequencing technology, post-clinical biopsy sample submission for pathogen culture seems to be the gold standard. However, it is also quite challenging. Although clinicians have made every effort to adhere to the principle of sterility when obtaining samples, it is still impossible to avoid contamination of the samples by external or endogenous microorganisms from surgical instruments, pathological section knives, laboratory environments, extraction reagents, library construction reagents, and even aerosols. These contamination issues are more common in the study of microorganisms in low-biomass samples. In batch effect experiments, systematic technical variations caused by non-biological factors such as experimental dates and reagent batches can distort the apparent differences in microbial community results, and the erroneous biological associations obtained can misjudge the biological differences between the tumor subtypes and treatment responses (85). Therefore, verification and correction strategies are particularly important: introducing controls in library construction to clearly identify background contamination, adding standard samples to detect technical deviations, ensuring the randomness and reproducibility of the experiment, and using biological information correction tools. In addition, there must be rigorous independent verification methods to truly separate the real tumor internal microbial signals from the complex technical background noise, including visualizing spatial localization of specific *in situ* PCR detection, independent library sequencing after selective genome expansion, expanding samples from multiple regions and centers, comparing with high-quality public data, establishing animal models and *in vitro* co-culture systems, or combining the metabolomic analysis of microbial genomes (86–88).

## 6 Summary

Although most current studies on tumor biology are still correlational and require more research to prove that specific bacterial communities are the “cause” rather than the “effect,” by reasonably intervening in this complex microbial ecosystem, and combining the genetic test report with the microbial group test report, and optimizing it through diet, probiotics, even FMT, we do have the potential to break through many current bottlenecks in tumor treatment, especially reversing the drug resistance of immune-related treatments, and thus achieving the maximum therapeutic effect centered on the host. Therefore, we propose the use of microbial intervention as a new treatment strategy to improve the outcome of tumor treatment and achieve precise regulation of tumors by targeting microorganisms in tumor medicine.
